# Potential Antidiabetic Effects of Seaweed Extracts by Upregulating Glucose Utilization and Alleviating Inflammation in C2C12 Myotubes

**DOI:** 10.3390/ijerph18031367

**Published:** 2021-02-02

**Authors:** Eunyoung Kim, Jiamei Cui, Inhae Kang, Guiguo Zhang, Yunkyoung Lee

**Affiliations:** 1 Department of Food Science and Nutrition, Jeju National University, Jeju 63243, Korea; sky90710@jejunu.ac.kr (E.K.); cuijiamei@jejunu.ac.kr (J.C.); inhaek@jejunu.ac.kr (I.K.); 2 Interdisciplinary Graduate Program in Advanced Convergence Technology & Science, Jeju National University, Jeju 63243, Korea; 3 Shandong Provincial Key Laboratory of Animal Biotechnology and Disease Control, College of Animal Sciences and Technology, Shandong Agricultural University, 61 Daizong Street, Taian City 271018, China

**Keywords:** seaweeds, antidiabetic, C2C12 myotubes, AMPK

## Abstract

Seaweed is known to have various health-promoting effects. However, the mechanisms underlying seaweed’s antidiabetic effects remain unclear. We investigated the potential antidiabetic effects of seaweed water extracts and further examined their mechanism(s) using C2C12 mouse skeletal muscle cells. Briefly, we screened the physiochemical properties of seven seaweed extracts by comparing the antioxidant and α-glucosidase inhibitory effects. Among them, three seaweed extracts, *Undaria pinnatifida* sporophyll (UPS), *Codium fragile* (CF), and *Gracilaria verrucosa* (GV), were selected for further testing of their possible antidiabetic effects with underlying mechanisms using C2C12 myotubes. Consistent with the superior α-glucosidase inhibition of the three seaweed extracts, the extracts also enhanced glucose utilization in myotubes compared to the control. The upregulated glucose uptake by the seaweed extracts was reversed by an AMP-activated protein kinase (AMPK) inhibitor, compound C, in the UPS- and CF-treated groups. Furthermore, all three seaweed extracts significantly promoted the phosphorylation of AMPK which was completely blocked by pretreating with compound C. In addition, all three extracts reduced lipopolysaccharide-simulated TNF-α production in C2C12 cells. Our results demonstrated that all three seaweed extracts exhibited antidiabetic properties through not only the inhibition of glucose absorption but also the promotion of glucose utilization. Moreover, the regulation of inflammatory cytokine production by the extracts suggested their potential anti-inflammatory property which might play a critical role in protecting insulin sensitivity in a chronic inflammatory state. Taken together, UPS, CF, and GV are a promising source to modulate the glucose absorption and utilization in muscle cells partially via the AMPK pathway.

## 1. Introduction

Diabetes mellitus (DM) is considered to be one of the most problematic public health issues, and its worldwide prevalence has more than doubled over the past 30 years. In particular, the incidence of DM is increasing more rapidly in Asian countries such as Korea, China, and India [1,2]. Type 2 DM (T2DM) is the most prevalent form of DM, which accounts for roughly 90% of all cases [3].

Treatment of DM is combined with diet and exercise therapy, but it is mainly dependent on medication to prevent multiple risk factors including hyperglycemia, hyperlipidemia, and hypertension that consequently lead to its complications. The current therapeutic agents for DM include α-glucosidase inhibitor (AGI), glucagon-like peptide (GLP)-1 agonist, dipeptidyl peptidase (DPP)-IV inhibitor, sulfonylureas, meglitinides, biguanides (metformin), thiazolidinediones (TZDs), and sodium-glucose cotransporter 2 (SGLT2) inhibitor which differently act on its intervention sites [4].

Some therapeutic alternatives include glycemic control through the modulation of various enzymes and mediators, such as AMP activated protein kinase (AMPK), a highly relevant cellular energy sensor for metabolic homeostasis regulation with particular relevance in regulating insulin sensitivity. AMPK can be activated by a variety of stimuli including cellular stress, exercise, and hormones. It has been shown that the activation of AMPK lowers blood glucose by increasing peripheral glucose uptake and upregulating the metabolism of glucose and fatty acids [4]. In addition, diabetic cardiovascular disease (CVD) which is the main cause of death for DM could be benefited by some antidiabetic drugs via AMPK-related signaling pathways. In fact, some diabetes treatments including metformin and SGLT2 inhibitors, have displayed cardiovascular benefits highly related to AMPK, although underlying mechanisms of the action are still uncertain [5]. Therefore, antidiabetic drugs targeting AMPK have been a focus of pharmaceutical companies [6,7].

Skeletal muscles are a primary tissue involved in glucose utilization in the human body. Therefore, these organs play a critical role in regulating glucose homeostasis. Principally, two pathways play roles in glucose metabolism in skeletal muscles: one is stimulated by phosphoinositide 3 kinase (PI3K) and the other is through AMPK. Insulin stimulates glucose uptake through insulin signaling pathways in skeletal muscle cells. On the other hand, AMPK is another important signaling molecule that enhances cellular glucose uptake independently of insulin [8]. AMPK is known to play a regulatory role in energy homeostasis and increasing glucose uptake into skeletal muscles [6].

The pharmaceutical approach to treating and managing patients with DM is efficient; however, long-term use of such drugs may have various side effects including toxicity and resistance [9]. Some therapeutic alternatives include glycemic control through the modulation of various enzymes and mediators, such as AMP activated protein kinase (AMPK), a highly relevant cellular energy sensor for metabolic homeostasis regulation with particular relevance in regulating insulin sensitivity in liver and muscle tissues. Therefore, antidiabetic drugs targeting AMPK have been a focus of pharmaceutical companies [6,7]. For example, thiazolidinediones and metformin are the standard drugs used to manage T2DM [6].

The pharmaceutical approach to treating and managing patients with DM is efficient; however, long-term use of such drugs may have various side effects including toxicity and resistance [9]. Accordingly, there has been enormous interest in developing drugs or diet therapies using naturally derived ingredients to minimize the side effects of long-term DM drug use [9,10]. For example, recent studies demonstrated that naturally occurring compounds such as resveratrol, ginsenoside, and curcumin were able to activate AMPK signaling and enhanced glucose metabolism by promoting intracellular glucose uptake [11,12,13].

Seaweeds have been consumed in various forms for many years in regions of Asia, including Korea. Seaweeds are a rich source of dietary fiber and contain small amounts of polyunsaturated fatty acids as well as bioactive substances such as essential amino acids, vitamins, minerals, and polyphenols [14,15]. It has been well-demonstrated that seaweeds have various effects that are beneficial for health, such as anti-inflammatory [16,17,18,19,20], antidiabetic [18,19,21,22,23,24], antiobesity [23,24,25,26], anticancer [20,27,28,29], and antimicrobial effects [30,31]. In fact, epidemiological studies have demonstrated that people of certain populations who consume high amounts of seaweeds tend to have a lower incidence of metabolic syndrome [32,33]. For instance, seaweeds are considered to be part of a healthy diet in Asia, and the countries of this part of the world have a lower prevalence of metabolic syndrome [34].

However, these studies did not include various types of seaweed. In addition, most of these cases were limited to brown seaweed, which is consumed more often than green and red seaweed. It is important to accumulate scientific evidence along with detailed analyses of the molecular mechanism(s) to effectively apply seaweed as a functional substance. Despite the numerous studies regarding the potential health-beneficial effects of seaweeds, its mechanism of antidiabetic action in skeletal muscle remains unclear. We hypothesized that seaweed extract regulates glucose homeostasis by regulating insulin signaling-related factors in skeletal muscle cells. In the present study, we investigated whether seaweed extract suppresses hyperglycemic conditions by regulating glucose uptake through the in vitro activation of Akt and/or AMPK signaling pathways. Briefly, we evaluated the antidiabetic and anti-inflammatory effects of seven seaweeds, including one green seaweed: *Codium fragile* (CF); four brown seaweeds: *Sargassum fulvellum* (SF), *Undaria pinnatifida* (UP), *Undaria pinnatifida* sporophyll (UPS), and *Ecklonia stolonifera* Okamura (ESO); and two red seaweeds: *Gelidium amansii* (GA) and *Gracilaria verrucosa* (GV). Based on the biochemical results, three seaweed extracts, UPS, CF, and GV, were then selected for further experiments toward establishing the potential mechanisms of antidiabetic action in C2C12 myotubes, mouse-derived skeletal muscle cells

## 2. Materials and Methods

### 2.1. Seaweed Water Extracts

Seaweeds (CF, SF, UP, UPS, ESO, GA, and GV) were purchased from a market in Jeju, South Korea, in 2016. The seaweed powders were then prepared by lyophilization followed by pulverizing prior to extraction. The specific details of powdered seaweed extract preparation are described elsewhere [18]. The obtained powdered seaweeds were prepared as solution using phosphate-buffered saline (Gibco, BRL, MD, USA) and used in the following experiments.

### 2.2. Alpha-Glucosidase Inhibitory Properties of Seaweed Extracts

Determination of α-glucosidase activity in the seaweed extracts was performed according to a previously reported method by Watanabe with some modifications [19,35]. Briefly, yeast α-glucosidase (0.7 U, Sigma, St. Louis, MO, USA) and 5 mM p-nitrophenyl-α-D-glucopyranoside (p-NPG, Sigma, St. Louis, MO, USA) were each prepared in 100 mM phosphate buffer (pH 7.0) for respective preparation of the enzyme and substrate solutions for the reaction. The 100 mM phosphate buffer contained 0.2% bovine serum albumin (Thermo Fisher Scientific, Waltham, IL, USA) and 0.02% NaN_3_ (Sigma, St. Louis, MO, USA). Then, 50 μL of enzyme solution was added to a 96-well plate containing 10 μL of seaweed extracts. After which, a substrate, 50 μL p-NPG was reacted with the enzyme solution for 5 min at room temperature, and the absorbance was measured at 405 nm by a microplate reader (Molecular Devices).

### 2.3. Total Polyphenol Levels of Seaweed Extracts

The total polyphenol contents of the seaweed extracts were calculated using the modified Folin–Denis method [36]. Firstly, 50 µL of 1 M Folin–Ciocalteu’s phenol reagent (FMD Millipore Corporation, Burlington, MA, USA) was added to 50 µL of seaweed extracts and allowed to stand at room temperature for 5 min. Then, 100 μL of 2% Na_2_CO_3_ solution was added to the reaction followed by incubation for another 30 min at room temperature in the dark. The absorbance was measured at 720 nm using a microplate reader (Molecular devices), and the total polyphenol contents were expressed as gallic acid concentration equivalents (GAE).

### 2.4. Free radical Scavenging Activities of Seaweed Extracts

The antioxidant properties of seaweed extracts were assessed using free radical assays, including 2,2′-azinobis-(3-ethyl-benzothiazoline-6-sulfonic acid) (ABTS) and 1,1′-diphenyl-2-picrylhydrazyl (DPPH) assays. The ABTS radical scavenging activity was measured according to the methods of ABTS radical cation decolorization assay with some modifications [37]. Briefly, ABTS was dissolved to a 7 mM concentration in water and mixed with a 2.6 mM potassium persulfate solution at a ratio of 1:1. Then, the mixture was allowed to stand in the dark at room temperature for 24 h for production of the ABTS radical cation. The working solution of the ABTS radical cation was prepared by dilution with distilled water to obtain an absorbance of 1.4–1.5 at 735 nm using a microplate reader (Molecular Devices). Thereafter, 25 μL of seaweed extracts were added to 190 μL of the ABTS radical cation working solution and incubated at 37 °C for 30 min. The absorbance was measured at 735 nm.

The DPPH radical scavenging activity was measured by previously described method with some modifications [37]. Briefly, DPPH was dissolved in ethanol to a 0.2 mM concentration. In a 96-well plate, 25 μL of the seaweed extracts and 175 μL of DPPH solution were incubated at 37 °C for 30 min, and the absorbance was measured at 517 nm. Ascorbic acid was used as a positive control. The free radical scavenging activity (ABTS, DPPH) was calculated according to the following formula:

Free radical scavenging activity (%) = [1 − (absorbance of sample/absorbance of control)] × 100.

### 2.5. Cell Culture and Differentiation

Mouse myoblast cell line, C2C12 (American-Type Culture Collection (ATCC), Manassas, USA) was cultured in high-glucose Dulbecco’s modified eagle medium (DMEM) containing 10% fetal bovine serum (FBS), 1% L-glutamine, and 1% penicillin-streptomycin (P/S) at 37 °C in a humidified atmosphere with 5% CO_2_. For differentiation into C2C12 myotubes, C2C12 myoblasts were seeded in either a 6-well plate or 96-well plate at 2.5 × 10^5^ or 0.5 × 10^5^ cells/well, respectively. When 90% to 100% confluence was reached, the medium was changed to DMEM supplemented with 2% horse serum and 1% P/S and differentiated continued for an additional 7 days. All materials used in the cell culture were purchased from Gibco.

In the experiments where the influence of compound C (an AMPK inhibitor, Sigma, St. Louis, MO, USA) on the effects of seaweed extracts was measured, cells were pretreated with or without 20 μM compound C for 30 min, followed by a treatment with seaweed extracts for 1 h.

### 2.6. Cell Viability

MTT assay was used to investigate the effect of extract of seaweeds on cell viability. C2C12 myoblasts were seeded into 96-well plates (0.5 × 10^5^ cells/well) and maintained in the growth medium to achieve 90–100% confluency. Then, 10% FBS was exchanged with 2% horse serum to induce differentiation over about 6 days. Seaweed extracts were treated at various concentrations (0–100 μg/mL) for 24 h. At the end of the treatment, the MTT solution was added and incubated for 3–4 h at 37 °C in humidified air and 5% CO_2_. For the viability assay, the formazan product was dissolved in 100 μL DMSO (dimethylsulfoxide) and the absorbance measured at 540 nm using a microplate reader (Molecular devices).

### 2.7. Determination of Glucose (2-NBDG) Uptake

Glucose uptake was analyzed by measurement of 2-[N-(7-nitrobenz-2-oxa-1,3-diazol-4-yl) amino]-2-deoxy-d-glucose (2-NBDG) uptake. C2C12 myoblasts were plated (1.0 × 10^5^ cells/well in a 12-well plate) to achieve 90–100% confluency. Furthermore, differentiation was induced for about 6 days. Differentiation-induced C2C12 cells (i.e., myotubes) were cultured in serum and glucose free DMEM medium for 3 h. The samples and 2-NBDG were treated with 100 μg/mL and 10 μM, respectively, and insulin (Sigma) was used as a positive control. C2C12 myotubes were rinsed with cold DPBS, and then added with 1% Triton-X-100 (Sigma, St. Louis, MO, USA). Fluorescence values were then measured at 485 nm and 528 nm using a microplate reader (Molecular Devices).

### 2.8. Western Blotting Analysis

To determine the alteration of AMPK activation by seaweed extracts in C2C12 cells, Western blotting analysis was performed as described previously [19]. Briefly, the extracted proteins (30~50 μg/24 μL) from the C2C12 myotubes treated with either control or seaweed extracts were quantified. Equal amounts of proteins were loaded and separated by SDS–PAGE and transferred to a nitrocellulose membrane. The membranes were incubated with the indicated antibody and horseradish peroxidase-coupled anti-species antibodies. The antibodies used were the following; phosphorylated AMPK (Thr172, Cell Signaling, Beverly, MA, USA, 1:1000), AMPK (Cell Signaling, Beverly, MA, USA, 1:1000), GAPDH (Cell Signaling, Beverly, MA, USA, 1:1000). Proteins were visualized using Chemidoc (Bio-Rad, Hercules, CA, USA) and quantified using Image J (National Institutes of Health, Bethesda, MD, USA).

### 2.9. Inflammatory Cytokine Detection by ELISA

C2C12 myotubes were pretreated with seaweed extracts in order to determine the amount of inflammatory cytokine production. After 3 h, the cells were stimulated with lipopolysaccharide (LPS) at a concentration of 100 ng/mL, cultured for 24 h, and all culture supernatants were then collected. The ELISA kit (BD PharMingen, San Jose, CA, USA) was used for the experiment according to the protocol. After measuring the inflammatory cytokine production in the media at the absorbance at 450–570 nm using a microplate reader (Molecular devices), the changes in tumor necrosis factor-α (TNF-α) and interleukin-10 (IL-10) production by seaweed extracts were calculated based on their standard curves.

### 2.10. Statistical Analysis

Statistical analyses were performed using *t*-testing or ANOVA (one-way analysis of variance and all data were reported as the mean ± standard error of the mean (SEM). Statistical significance was set at *p* value < 0.05. GraphPad Prism software (Version 8.0.1, San Diego, CA, USA) was utilized for all data analysis.

## 3. Results

### 3.1. Alpha-Glucosidase Inhibitory Activity of Seven Seaweed Extracts

**Figure 1:**
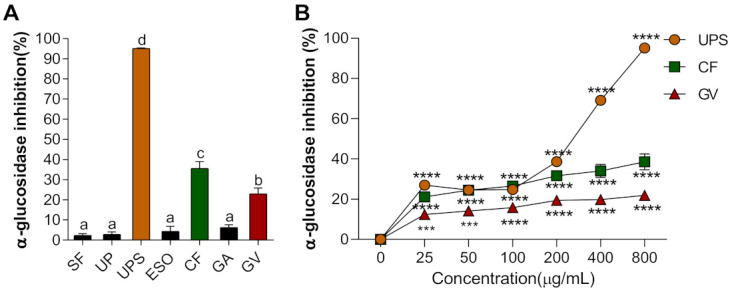
(**A**) Alpha-glucosidase inhibition rate of seven different seaweed extracts. Values that do not share the same superscript are significantly different according to ANOVA (*p* < 0.05). (**B**) α-glucosidase inhibition rate of three selected seaweed extracts at various concentrations. All values are presented as the mean ± SEM of three independent experiments (*n* = 3–4/group for each experiment); *** *p* < 0.001, **** *p* < 0.0001 compared with control group (0 μg/mL) using one-way ANOVA with Dunnett’s comparison test. Abbreviations: SF, *Sargassum fulvellum*; UP; *Undaria pinnatifida*; UPS, *Undaria pinnatifida* sporophyll; ESO, *Ecklonia stolonifera* Okamura; CF, *Codium fragile*; GA, *Gelidium amansii*; GV, *Gracilaria verrucosa.*

Alpha-glucosidase is a carbohydrate hydrolysis enzyme to control postprandial blood glucose levels, therefore its inhibitory property has been commonly used for screening natural products for their potential antidiabetic property [38]. We evaluated extracts of seven seaweed extracts that may be useful for diabetic treatment by delaying glucose absorption. The rate of inhibition of α-glucosidase activity was the highest in UPS (96.13%) extracts, followed by CF > GV > GA, ESO, and SF, as shown in Figure 1A. In addition, for the top three extracts (UPS, CF, and GV), the inhibition of α-glucosidase activity appeared to be dose-dependent (Figure 1B).

### 3.2. Total Polyphenol Contents and Antioxidant Properties of Three Seaweed Water Extracts

**Figure 2:**
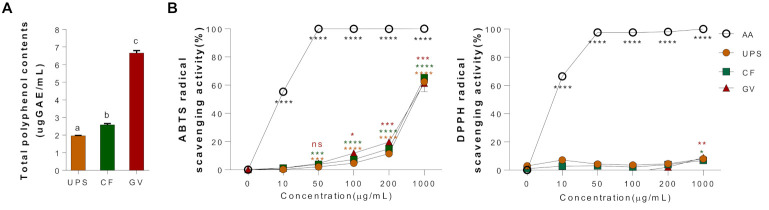
Total polyphenol contents and antioxidant properties of three seaweed water extracts. (**A**) Total polyphenol contents, values that do not share the same superscript are significantly different according to ANOVA (*p* < 0.05), (**B**) ABTS cation radical scavenging effect and DPPH cation radical scavenging effect of UPS, CF, and GV. Data are represented as the mean ± SEM of three independent experiments. * *p* < 0.05; ** *p* < 0.01, *** *p* < 0.001, **** *p* < 0.0001 compared with control group (0 μg/mL) using one-way ANOVA with Dunnett’s comparison test. Abbreviations: AA; ascorbic acid, UPS, *Undaria pinnatifida* sporophyll; CF, *Codium fragile*; GV, *Gracilaria verrucosa.*

Total polyphenol contents of three seaweed water extracts (UPS, CF, and GV) were examined, as shown in Figure 2A. The content of polyphenol compounds was expressed as μg of gallic acid equivalents (GAE). GV extract appeared to have the highest polyphenol contents (6.33 ± 0.20 μgGAE/mL) among the three different seaweed extracts, followed by CF (2.58 ± 0.03 μgGAE/mL) and UPS (1.97 ± 0.04 μgGAE/mL). In addition, we evaluated the antioxidant properties of three seaweed extracts by measuring the ABTS and DPPH radical scavenging activities. Figure 2B shows the free radical scavenging activity in a dose-dependent manner of the seaweed extracts presented as a percentage of the ratio of the decrease in absorbance of the test solution to that of ABTS solution without the seaweed extracts. On the other hand, the three seaweed extracts did not have the DPPH radical scavenging activity.

### 3.3. Cellular Toxicity

**Figure 3:**
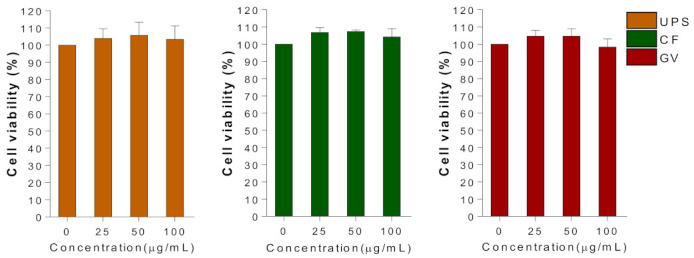
Effect of the three seaweed extracts on cell viability in C2C12 myotubes. Differentiated C2C12 cells were treated with three types of seaweed extracts for 24 h and MTT assay was performed as described in methods. Data are represented as the mean ± SEM of three independent experiments. Abbreviations: UPS, *Undaria pinnatifida* sporophyll; CF, *Codium fragile*; GV, *Gracilaria verrucosa.*

In order to determine the optimal concentrations of the selected three seaweed extracts to conduct further in vitro studies, the viability of C2C12 myotubes was evaluated at a dose range of 0–100 μg/mL using MTT assay (Figure 3). Within the tested concentrations of UPS, CF, and GV, 100 μg/mL of seaweed extract was used in subsequent experiments.

### 3.4. Mechanism of Antidiabetic Action by the Seaweed Extracts in C2C12 Myotubes

**Figure 4:**
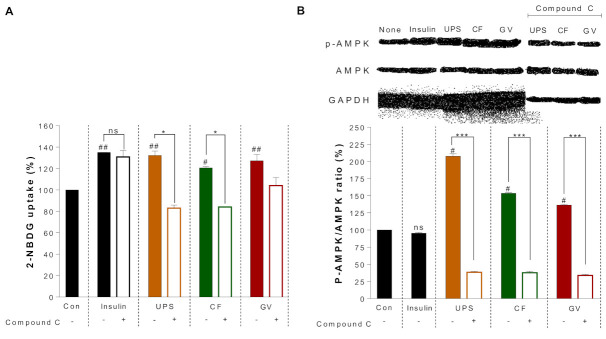
Effects of three seaweed extracts on glucose (2-NBDG) uptake and phosphorylation of AMPK with or without compound C in C2C12 myotubes. Myotubes were treated with 20 μM of compound C and then exposed to 100 μg/mL of the three seaweed extracts. (**A**) Alteration of 2-NBDG uptake rate by seaweed extracts with or without compound C, # *p* < 0.05, ## *p* < 0.01; compared with control using one-way ANOVA with Dunnett’s comparison test, * *p* < 0.05; compared values with and without compound C using *t*-test, (**B**) alteration of AMPK phosphorylation by UPS, CF, and GV treatment with or without compound C in C2C12 myotubes. # *p* < 0.0001; compared with control using one-way ANOVA with Dunnett’s comparison test, * *p* < 0.05, *** *p* < 0.0001 compared values with and without compound C using *t*-test. Data are represented as the mean ± SEM. Abbreviations: UPS; *Undaria pinnatifida* sporophyll, CF; *Codium fragile*, GV; *Gracilaria verrucose*, Control; Con.

For the glucose uptake assay, C2C12 myotubes were incubated with a fluorescent D-glucose analogue, 2-NBDG, which was used as a tracer to monitor glucose uptake in C2C12 myotubes. As shown in Figure 4, a stimulatory effect on glucose uptake caused by UPS, CF, and GV was observed in C2C12 myotubes. Compared to the control, all three seaweed extracts significantly increased the glucose uptake. UPS, CF, and GV increased glucose uptake by 132.2% ± 5.73%, 120.6% ± 1.46%, and 127.2% ± 8.58%, respectively, which was comparable to the action of insulin. In addition, to determine whether AMPK is involved in the glucose uptake effect of the seaweed extracts, we attempted to inhibit AMPK activity using a pharmacological approach. Pretreatment of C2C12 myotubes with compound C, an AMPK inhibitor, significantly attenuated CF and GV-induced 2-NBDG uptake. However, treatment with a PI3K inhibitor (LY294002) did not affect 2-NBDG absorption induced by the seaweed extracts (data not shown). Next, we further investigated whether the seaweed extracts itself could activate AMPK by increasing phosphorylation. As shown in Figure 4B, UPS, CF, and GV indeed increased AMPK phosphorylation in C2C12 myotubes which was completely blocked by pretreatment with compound C, suggesting that insulin-independent AMPK activation was involved in the glucose uptake by the three seaweed extracts.

### 3.5. Immunomodulating Effects of the Three Selected Seaweed Extracts in C2c12 Myotubes

**Figure 5:**
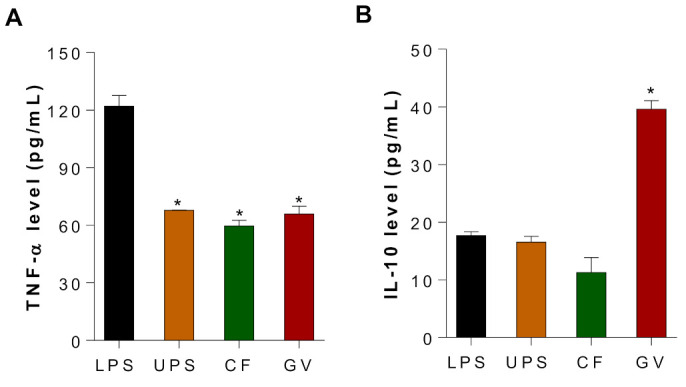
Effects of three seaweed extracts on TNF-α and IL-10 production in LPS-stimulated C2C12 myotubes. (**A**) TNF-α and (**B**) IL-10 production after UPS, CF, and GV treatment of C2C12 myotubes. C2C12 myotubes were pretreated with seaweed extract for 3 h, and LPS (100 ng/mL) was added for another 24 h. Data are represented as the mean ± SEM. Asterisks represent statistical significances compared with values of LPS treated group according to ANOVA (*p* < 0.05). Abbreviations: LPS, Lipopolysaccharide; UPS, *Undaria pinnatifida* sporophyll; CF, *Codium fragile*; G*V*, *Gracilaria verrucosa.*

To demonstrate the potential anti-inflammatory effects of UPS, CF, and GV water extracts in C2C12 myotubes, LPS-stimulated C2C12 myotubes were treated with three different seaweed extracts, and production of TNF-α and IL-10 were measured using ELISA. The expression of TNF-α, an inflammatory cytokine associated with chronic inflammation and insulin resistance (IR), decreased in the three seaweed groups as compared to the LPS stimulated group (Figure 5A). In addition, LPS-induced IL-10, an anti-inflammatory cytokine, was further increased by GV treatment in C2C12 myotubes, whereas UPS and CF did not alter the IL-10 production (Figure 5B). It should be noted that both cytokines were not detectable in the negative control group where LPS was not treated in C2C12 myotubes.

## 4. Discussion

DM, and especially T2DM, is a chronic metabolic disorder with worldwide prevalence. T2DM is characterized by IR and hyperinsulinemia, which lead to high blood glucose levels and vascular complications [21]. The incidence of such complications can be reduced by lowering blood sugar levels [39]. Hence, for effective management of T2DM, it is crucial that we find ways to promote glucose uptake in skeletal muscle and reduce glucose production in the liver [39,40]. Recently, many types of medicinal plants and their bioactive compounds have been reported to increase insulin sensitivity, thus leading to the emergence of renewed interest in alternative medicines and natural therapies to treat T2DM. Seaweeds, inter alia, have evolved the capability to grow in extreme environments (i.e., high pressure, salinity, and temperature). Therefore, seaweeds contain abundant bioactive compounds that are not found in terrestrial plants [41].

Alpha-glucosidase is located in the brush border surface membrane of intestinal cells, and this enzyme activates the final step of the digestion [42], therefore it has been considered as the main target in the prevention and treatment of T2DM. Currently, there are various antidiabetic drugs (i.e., acarbose, miglitol, and voglibose) that inhibit α-glucosidase activity. However, the continuous use of these drugs is often associated with undesirable side effects, such as gastrointestinal disorders. Therefore, there is a need for natural α-glucosidase inhibitors that have no associated toxicity (liver or kidney) or unwanted side effects [42,43]. In this study, among seven tested seaweed extracts, UPS, CF, and GV water extracts significantly appeared to have potent inhibitory effects on α-glucosidase activity among seven different seaweed extracts, and their inhibitory property was dose-dependent. In general, seaweeds are considered to be a rich source of antioxidants [14,15,44,45]. Antioxidant activity is defined as the ability of a compound to inhibit oxidation decomposition, such as lipid peroxidation [46,47]. The potential antioxidant compounds in these seaweeds have been identified as pigments (i.e., fucoxanthin and carotenoid) and polyphenols (i.e., flavonoid, phenolic acid, and tannins) [24]. Thus, the polyphenols contained in this natural product are suspected to be responsible for some of its pharmacological effects. For example, Bu et al. reported that phenolic compound, butyl-isobutyl-phthalate isolated from brown seaweed, *Laminaria japonica*, had α-glucosidase inhibitory property [48]. In addition, polyphenols from jute leaf, *Corchorus olitorius*, also demonstrated the inhibitory effects on α-glucosidase [49] In this study, GV extract appeared to have the highest polyphenol content among the three different seaweed extracts, followed by CF and UPS which was not well-reconciled with their α-glucosidase inhibitory as well as antioxidant properties. That is, GV extract having the highest polyphenol contents did not present the strongest α-glucosidase inhibitory property among three seaweed extract. Further chemical analysis such as phenolic compounds in the seaweed extracts could give us better understanding about this inconsistency. Moreover, we showed that the three seaweed extracts (UPS, CF, and GV) exhibited a significant ABTS radical scavenging activity, but no DPPH radical scavenging activity. It should be noted that each component is differed in their capabilities to scavenge a type of radical. A previous study by Kim et al. showed similar phenomenon when they performed the ABTS and DPPH radical scavenging activities of 10 types of seaweeds [50].

Suppressed insulin signaling or IR in skeletal muscle cells is one of the characteristic features of T2DM. The amount of glucose uptake by skeletal muscles constitutes the majority of that taken up by the whole body (more than 80%) [51,52]. Therefore, skeletal muscles perform an essential function in maintaining glucose homeostasis; thus, an ideal antidiabetic therapy must effectively affect muscle glucose intake. In this study, all three seaweed extracts (UPS, CF, and GV) significantly enhanced glucose uptake in C2C12 myotubes. Insulin signaling for promotion of glucose uptake in skeletal muscles is initiated by activating PI3K and Akt [8]. Another glucose utilization related enzyme, AMPK, promotes intracellular glucose uptake, though its regulation occurs independently of insulin. It is well documented that AMPK is activated by exercise and antidiabetic drugs such as metformin, as well as various phytochemicals [6,8,53]. Metformin is an oral glucose-lowering agent which has been widely used to treat T2DM. The main effect of metformin is to improve hyperglycemia by increasing glucose utilization and reducing hepatic glucose production. Recent studies showed that a variety of natural products have effects on AMPK activation similarly to metformin [54,55]. Our findings also demonstrated that glucose uptake and AMPK phosphorylation were significantly enhanced by UPS and CF, and the upregulation was reversed by an AMPK inhibitor suggesting that AMPK could be a factor in regulating the glucose uptake by certain seaweed extracts.

Whole seaweeds or bioactive compounds isolated from seaweeds, which can promote glucose uptake into muscle cells and subsequently improve muscle IR, will be useful in T2DM management [21]. For this reason, several seaweed species have been investigated for their ability to induce glucose uptake into cells and tissues, with the aim of reducing blood glucose levels and hyperglycemia. Our previous study demonstrated that application of extracts of the brown seaweed, *Laminaria japonica* (LJ) and *Hizikia fusiforme* (HF), significantly increased glucose uptake in C2C12 myotubes. Furthermore, it was confirmed that Akt and AMPK were significantly activated in C2C12 myotubes and diet-induced obese mice tissues by LJ and HF supplementation, respectively [19]. Interestingly, the activation of insulin signaling pathways such as Akt and AMPK by brown seaweed extracts in C2C12 myotubes was similarly observed in the skeletal muscles of mice fed high fat diet supplemented with 5% freeze-dried LJ or HF. In another study, Kang et al. attributed a reduction in postprandial blood sugar levels to the AMP-activated protein kinase/ACC and PI-3K/Akt signaling pathways in C2C12 myotubes and streptozotocin-induced diabetic mice to supplementation with the brown seaweed (*Ecklonia cava*) extract [56]. Therefore, it is important to note that the enhancement of glucose regulation in muscle by seaweed extracts may be modulated in a species-specific manner even among brown seaweeds.

IR is highly associated with obesity and inflammation [57]. Although obesity-related inflammation is relatively less studied in muscle than in adipose tissue, several studies reported the capacity of muscle producing a number of inflammatory cytokines [58]. In obese state, lipid accumulation also occurs in muscle similarly to adipose tissue which ultimately contributes to IR [58]. Thus, infiltrated immune cells and inflammatory activation induce inflammation in skeletal muscle in obesity. The cytokines’ secretion by muscle are regulated by various factors such as muscle contraction, glucose and lipid metabolism, and inflammation [58,59]. In our study, we showed that all three types of seaweed (UPS, CF, and GV) significantly ameliorated TNF-α production in LPS-stimulated C2C12 myotubes, whereas GV promoted IL-10 production showing differential regulation of three seaweed extracts in immunomodulatory properties. A similar observation was also reported in our previous study, in which LJ and HF water extracts significantly decreased TNF-α protein levels and promoted IL-10 protein levels in LPS-stimulated C2C12 myotubes [19]. In addition, Khan et al. demonstrated that application of methanolic extracts of a brown seaweed, *Undaria pinnatifida*, and green seaweed, *Ulva linza*, inhibited inflammatory responses in BALB/c mice indicated by inhibited edema and erythema [60]. Furthermore, fucoxanthin, a carotenoid found in brown seaweeds, downregulated the expression of proinflammatory cytokines such as MCP-1, TNF-α, and IL-6, both in vivo and in vitro [61]. It is well documented that hypersecretion of proinflammatory cytokines has been implicated in pathogenesis of IR and DM complications [59]. Several studies reported that proinflammatory cytokines play an important role in regulating glucose homeostasis. Therefore, understanding the exact mechanisms of insulin resistance and inflammatory cytokines in skeletal muscle, a major tissue for glucose metabolism, may help to develop new treatments that rectify glucose metabolism [58,62]. It should be noted that the phenomena observed here were restricted to seaweeds extracted with water, which could differ from seaweed extracts prepared with other solvents. This study has several limitations: (1) the responsible component(s) in the extractions were not identified and remain to be elucidated, and (2) the actual muscle glucose uptake system could not be completely mimicked in C2C12 myotubes. Future work will be warranted whether the potential antidiabetic effects of UPS, CF, and GV targeting muscle cells could benefit insulin sensitivity and local and systemic chronic inflammation in a diet-induced obese mouse model. Moreover, their bioactive compounds require further investigation.

In spite of the limitations mentioned above, our work provides additional insights into potential antidiabetic property and partial mechanism of the three seaweeds, UPS, CF, and GV with respect to inhibiting α-glucosidase activity, promoting glucose uptake and AMPK activation, as well as anti-inflammatory effect in muscle cells.

## 5. Conclusions

The present study demonstrated that seven different seaweed extracts had α-glucosidase inhibitory properties and antioxidant activities. Among them, three seaweed extracts (UPS, CF, and GV) were further examined. In summary, seaweed water extracts from UPS, CF, and GV positively affect glucose uptake metabolism and inflammatory response in C2C12 myotubes. Our results suggest that some seaweed have potential in regulating and activating the AMPK-dependent pathway in C2C12 myotubes, which can lead to managing diabetes mellitus and the related complications. Hence, we suggest that seaweed, UPS, CF, and GV, could be potential antidiabetic agent to improve muscle IR and the associated inflammation.

## Data Availability

Not applicable.
